# Prophylaxis in ant societies: a social response to pathogen vulnerability?

**DOI:** 10.1098/rsos.261179

**Published:** 2026-11-11

**Authors:** Romain Willeput, Claire Detrain

**Affiliations:** Unit of Social Ecology, Université Libre de Bruxelles, Brussels, Belgium

**Keywords:** *Lasius flavus*, *Beauveria bassiana*, social immunity, necrophoresis, grooming behaviour, pathogen susceptibility

## Abstract

Social insects rely on multiple defence mechanisms to limit pathogen transmission within colonies. Prophylactic sanitary behaviours are performed in the absence of immediate pathogenic threats and contribute to routine disease prevention. Among these behaviours, grooming and necrophoresis help manage potentially harmful biological material, but how their expression relates to variation in pathogen susceptibility among colonies remains poorly understood. We investigated the relationship between susceptibility to the entomopathogenic fungus *Beauveria bassiana* and the expression of prophylactic grooming and necrophoresis in the ant *Lasius flavus*. We found strong and consistent intercolonial differences in pathogen susceptibility, as well as marked variation in the expression of prophylactic sanitary behaviours. None of the grooming behaviours examined were associated with pathogen susceptibility. However, more pathogen-susceptible colonies tended to remove corpses more rapidly during the early phase of necrophoresis. This pattern is consistent with a possible compensatory relationship among defence components, but may also arise from demographic and organizational differences among colonies, as worker age structure and task allocation can influence both susceptibility to infection and corpse removal behaviour. Overall, our results reveal substantial intercolonial variation in multiple components of disease defence but provide little support for a compensatory relationship between pathogen susceptibility and prophylactic sanitary behaviour.

## 1. Introduction

Pathogens represent a major selective pressure shaping the ecology and evolution of animal populations. This pressure can be particularly pronounced in animal societies, where individuals live in dense and highly interactive groups sharing a common nest, thereby facilitating pathogen transmission [1,2]. Social insects such as ants form large colonies composed of numerous closely related individuals, creating conditions that may promote rapid disease spread and shared susceptibility of nestmates to the same pathogens [3]. To counter these threats, insect societies have evolved a wide array of individual and social immune defences that reduce pathogen exposure and transmission within colonies, making large-scale disease outbreaks relatively rare in natural populations [4].

In ants, these defences encompass physical barriers, immune responses and a range of behavioural defences. The cuticle acts as a first barrier against contact-transmitted pathogens, while cellular and humoral immune responses inhibit pathogen growth once it has entered the insect body [5,6]. Ant workers also employ behavioural defences that reduce pathogen load on the body surface. Self-grooming allows workers to remove infectious particles before they penetrate the cuticle. Workers may also groom their nestmates (allogrooming), thereby helping to eliminate pathogens from the bodies of other colony members [7,8]. Furthermore, during grooming, ants can spread antimicrobial compounds from exocrine glands across the cuticle, thereby reducing the likelihood of infection [9,10]. In addition to grooming, ant societies rely on other sanitary behaviours that reduce pathogen exposure within the colony [11]. A key component of these sanitary defences is the management of potentially hazardous items within the nest, such as waste materials, contaminated food, diseased individuals or corpses [12]. Because these items may harbour pathogens or promote microbial growth, their accumulation can increase the risk of infection among densely aggregated nestmates [13]. Consequently, many social insects actively remove or spatially segregate waste from the brood and the main nest chambers. Necrophoresis, the removal of dead nestmates from the nest, is one of the most widespread sanitary responses observed in ants [14,15]. Corpses represent potential reservoirs of microorganisms and may therefore constitute an important source of pathogens. The rapid detection and removal of corpses from the nest are thus thought to play an important role in limiting disease spread and maintaining colony hygiene [16–18].

Different components of disease defence do not necessarily operate independently, as behavioural and physiological defences can interact and jointly shape protection against infection [19–21]. Across individuals or colonies, these components can be positively related, negatively related or unrelated. Because immune defences entail energetic and physiological costs, natural selection may favour different combinations of defence mechanisms, potentially leading to evolutionary trade-offs [22,23]. Accordingly, stronger expression of one defence component may be associated with weaker expression of another. In subsocial burying beetles, challenging personal immunity reduces the antibacterial activity of anal exudates used to sanitize the breeding carcass [24]. Similarly, in termites, weaker innate immunity is compensated by stronger allogrooming [25]. Conversely, different defence components may be positively associated, as pathogen-induced alarm behaviour is positively correlated with allogrooming in termite workers exposed to fungal pathogens [26]. Finally, different defence components can vary independently, with no relationship found between venom and haemolymph antimicrobial activities in bumblebees [27]. Overall, relationships among different components of disease defence appear to be highly variable and remain poorly understood.

While pathogen-induced sanitary behaviours have been widely studied, less is known about prophylactic sanitary behaviours, which contribute to routine disease prevention in the absence of pathogen exposure. In social insects, this includes the removal of corpses from the nest even when death results from non-infectious causes, thereby reducing potential future sanitary risks within the colony. Between-colony variation in such prophylactic corpse removal has been documented in both ants [28] and honeybees [29,30]. Because colonies differ in their susceptibility to pathogens [31], limiting sanitary risks through prophylactic behaviours may be particularly beneficial when susceptibility is high. Available evidence linking pathogen susceptibility to prophylactic removal of dead colony members remains limited and inconsistent. Cassidy *et al.* [28] found that ant colonies with greater susceptibility to fungal infection removed corpses more rapidly, a pattern they suggested could reflect a trade-off between individual and social immunity. By contrast, Harpur *et al.* [30] found no relationship between removal of dead brood and innate immunity in honeybees. Grooming is likewise expressed in the absence of direct pathogen exposure and may therefore contribute to routine prophylactic hygiene [32]. However, the relationship between intercolonial variation in prophylactic grooming and pathogen susceptibility has received little attention. Whether pathogen susceptibility covaries with the expression of prophylactic sanitary behaviours therefore remains unclear.

In this study, we investigated the relationship between pathogen susceptibility and prophylactic sanitary behaviours in the yellow meadow ant *Lasius flavus*. We first assessed intercolonial variation in susceptibility to the entomopathogenic fungus *Beauveria bassiana* and the repeatability of these between-colony differences in susceptibility. We then quantified colony-level expression of prophylactic grooming behaviours by measuring allogrooming, self-grooming and acidopore grooming. We also examined prophylactic corpse removal by monitoring the dynamics of necrophoresis following the introduction of non-infectious ant corpses into experimental nests. Finally, we tested whether colonies that differed in their susceptibility to fungal infection also differed in their prophylactic behavioural responses. From an evolutionary perspective, selection favouring stronger prophylactic behaviours could be accompanied by relaxed selection on costly innate immune mechanisms, potentially leading to their reduction over time [30]. We therefore expect both pathogen susceptibility and prophylactic behaviours to vary among colonies and predict that higher pathogen susceptibility will be associated with stronger expression of prophylactic behaviours. Such a pattern would be consistent with a compensatory relationship between different components of disease defence.

## 2. Methods

### 2.1. Collection and maintenance of ant colonies

*Lasius flavus* is a common Palaearctic ant species with a predominantly subterranean lifestyle. Colonies generally contain several thousand workers and rely largely on honeydew produced by root aphids living on herbaceous plants. The species occurs in a variety of open habitats, including grasslands and forest edges [33]. Seventeen colonies of *L. flavus* were collected in the vicinity of Ostend, Belgium, during the summer of 2024 (supplementary material, table S1). Although total colony size was not precisely quantified, thousands of workers were collected from each colony, but no queens. Nests were located in sandy, sun-exposed soil bordering hiking paths, a habitat characteristic of this species’ subterranean nesting preferences. Colonies were separated by a minimum distance of 10 m to prevent resampling of the same nest.

Each colony was transferred to a plastic container (40 × 25 × 7 cm) whose inner walls were coated with polytetrafluoroethylene (Fluon) to prevent worker escape. Ants were maintained under controlled laboratory conditions at 18 ± 2°C, 50 ± 5% relative humidity, and a 12:12 h light:dark photoperiod. Nests consisted of glass test tubes half-filled with water and plugged with cotton wool to provide a stable humid microenvironment. Test tubes were covered with red transparent film to replicate the darkness of natural nest chambers. In the foraging arena, workers had *ad libitum* access to water and a 0.3 M sucrose solution, and were provided with crickets (*Acheta domesticus*) once per week as a protein source.

### 2.2. Colony-specificity of pathogen susceptibility

The first experiment aimed to characterize intercolonial variation in pathogen susceptibility and to determine whether such differences were consistent across repeated infection assays. To quantify colony-level susceptibility to fungal infection, we measured the survival of *L. flavus* workers following exposure to *B. bassiana*. The pathogen was applied using Naturalis (CBC Europe), an oil-based commercial formulation containing viable conidia of *B. bassiana* strain ATCC 74040, at a concentration of 2.3 × 10⁷ CFU ml^–1^. Each worker was briefly immobilized with wide-tipped forceps, and a 1 µl droplet of the conidial suspension was deposited dorsally on the gaster using a micropipette, avoiding contact with the legs to preserve normal mobility of the treated ant. This inoculation corresponds to approximately 2.3 × 10⁴ CFU per individual. Control individuals received a 1 µl droplet of carrier oil alone, obtained by centrifuging the Naturalis formulation three times at 6000 rpm, removing the conidia pellet and retaining the supernatant. We assessed pathogen susceptibility of each of the 17 *L. flavus* colonies by exposing 20 workers per colony (10 intranidal workers and 10 foragers) to *B. bassiana* spores. To evaluate the temporal consistency of colony-level susceptibility, this procedure was repeated three times at one-week intervals, each time using a new set of workers. In total, 60 individuals were tested per colony (30 intranidal workers and 30 foragers). In each assay, an additional 10 workers per colony (5 intranidal workers and 5 foragers) served as controls to verify that mortality associated with the carrier oil, handling procedure and experimental conditions remained low. Foragers were collected directly at the food source, whereas intranidal workers were obtained by gently emptying nest tubes into a separate container and sampling individuals at random. Following treatment, each worker was isolated in a 35 mm Petri dish provided with two cotton wicks: one soaked in water and the other in 0.3 M sucrose solution. Mortality was recorded daily for 12 days. To confirm fungal-induced death, corpses were surface-sterilized in ethanol and rinsed with distilled water following Lacey's method [34]. They were then placed in sterile Petri dishes on moist filter paper, sealed with Parafilm, and incubated at 25°C until sporulation occurred (typically within 3–7 days).

### 2.3. Intercolonial variation in prophylactic grooming behaviour

The second experiment, conducted approximately one month after the start of the first infection assay, quantified the expression of prophylactic grooming behaviours in the absence of pathogen exposure. We measured allogrooming, self-grooming and acidopore grooming in workers from each of the 17 *L. flavus* colonies used in the survival assays. We focused on intranidal workers because they usually remain within the nest, where they interact with other colony members and participate in tasks related to nest hygiene. For each colony, five groups of five intranidal workers were collected and placed in 35 mm diameter Petri dishes whose inner walls were coated with Fluon to prevent escape of workers. Groups were kept in red-light chambers to mimic nest darkness and were allowed to acclimate for 10 minutes before their behaviour was recorded from above for 5 minutes. Given the low frequency of allogrooming, its cumulative duration (in seconds) was quantified over the entire 5-minute trial for the five workers in each group. By contrast, self-grooming occurred very frequently, making continuous quantification of its duration impractical. It was therefore assessed using a scoring approach: during the last 10 seconds of each minute, we counted how many of the five workers were engaged in self-grooming. These five counts (one per minute) were summed to generate a self-grooming score ranging from 0 to 25 per tested group. Acidopore grooming was quantified as the number of occurrences observed during the 5-minute video recordings, using each mouth-acidopore contact as a clearly identifiable behavioural event. For each colony, the median value across the five groups was used as a colony-level estimate for each grooming behaviour.

### 2.4. Intercolonial variation in corpse removal

The third experiment was conducted in parallel with the grooming experiment and investigated the dynamics of necrophoresis, the collective removal of non-infectious corpses introduced into the nest. For each of the 17 *L. flavus* colonies, a standardized queenless and broodless experimental subcolony of 500 workers was created by manually collecting 250 workers from the nest tubes and 250 workers from the foraging area of the colony. These workers were transferred into a plastic tray (40 × 25 × 7 cm) whose inner walls were coated with Fluon to prevent escape. The bottom of each tray was lined with a 2 cm layer of plaster, which acted as a humidity buffer and ensured constant hydration of the artificial nest. Workers occupied a circular nest (8 cm diameter, 1 mm height), covered with a transparent ceiling overlaid with red film and consisting of a single chamber with a single 1 cm-wide entrance. The low height of the chamber constrained workers to a single layer, thereby enabling unobstructed video recording of their behaviour. A vertical plastic tube plugged with cotton was inserted through a small opening in the nest roof to permit the introduction of corpses into the centre of the nest while temporarily preventing workers’ access to the corpses prior to the start of the experiment. Experimental subcolonies were provided *ad libitum* with water and a 0.3 M sucrose solution. They were also fed crickets one day before the trial to reduce the likelihood of workers feeding on the introduced corpses. These subcolonies were allowed to acclimate in the experimental nests for one week before the experiment. During this acclimation period, they were maintained under a 12:12 h light:dark photoperiod, with low ambient light during the light phase to encourage workers to settle beneath the artificial nest. On the day before the experiment, colonies were transferred to the recording chambers under red light, where they remained throughout the experiment.

Corpses used in the necrophoresis experiment were obtained by randomly collecting and freezing healthy *L. flavus* workers (–18°C for 2 hours). After freezing, each corpse was marked on the head with alcohol-based paint (Edding 750) to ensure unambiguous identification during video analysis. Preliminary trials assessing the influence of corpse age on necrophoresis in *L. flavus* showed that the proportion of corpses removed was highest at 24, 48 and 72 h post-mortem. Corpse age was therefore standardized at 48 hours. Corpses were stored for 48 hours in sealed Petri dishes at 18°C and high humidity (approx. 100% RH), allowing post-mortem chemical changes associated with corpse recognition to develop [35]. Five minutes before the start of the experiment, corpses were removed from the humid chamber to dry. Then, 20 corpses were gently placed inside the vertical insertion tube positioned into the central opening of the nest ceiling. The experiment started once the insertion tube was carefully removed from the nest, providing workers with access to the corpses. The opening in the nest ceiling was sealed immediately after tube removal to prevent workers from exiting through the nest roof. The nest interior and a 5 cm area surrounding the nest entrance were recorded for 4 hours. From these recordings, we continuously monitored the dynamics of corpse removal throughout the assay. Experiments were carried out under red light in order to mimic the darkness of natural nest conditions. To assess colony-level efficiency of necrophoresis, we used the area under the curve (AUC), which represents the cumulative residence time of corpses within the nest. AUC was calculated over the first 2 hours of the experiment to characterize early corpse removal and over the complete 4-hour observation period to characterize overall corpse removal. Lower AUC values indicate faster corpse removal, resulting in shorter cumulative exposure of colony members to corpses.

### 2.5. Statistical analyses

Analyses were performed in R version 4.3.1. We applied a significance threshold of α = 0.05 for all statistical tests. Median values are reported in the text in the format: median [first quartile; third quartile].

Worker survival across the 17 *L. flavus* colonies was analysed using a Cox proportional hazards model (*survival* package), with colony identity included as a fixed effect. Individual time-to-death data from the three experimental assays were analysed jointly, with assay included as a stratification factor to account for potential differences in baseline mortality among experimental sessions. Survival analyses were restricted to pathogen-exposed workers, as the objective was to quantify intercolonial variation in susceptibility following fungal exposure and mortality among control workers was very low. Differences in survival among colonies were assessed by comparing the full model to a null model using a likelihood ratio test (LRT). The proportional hazards assumption was examined using a Schoenfeld residual test and corresponding plot, which did not indicate evidence of a time-dependent colony effect (*p* = 0.058). To evaluate whether pathogen susceptibility was a consistent and colony-specific trait, the repeatability of mortality was quantified across the three infection assays. Repeatability was assessed using an intraclass correlation coefficient (ICC; *irr* package) calculated from the proportion of workers that had died by the end of the 12-day monitoring period in each colony and assay. The ICC estimated the proportion of total variance in mortality attributable to differences among colonies rather than to variation across assays, thereby quantifying the consistency of colony-level susceptibility to fungal infection. To test whether survival differed within colonies between functional groups of *L. flavus* workers (foragers versus intranidal workers), worker group was included as a fixed effect in a mixed-effects Cox proportional hazards model (*coxme* package), with colony identity included as a random effect to account for non-independence among workers originating from the same colony. Assay was again included as a stratification factor. The worker-group effect was assessed from the corresponding coefficient in the model using a Wald test, and its effect size was expressed as a hazard ratio (HR) with a 95% confidence interval. The Schoenfeld residual test and plot showed no evidence of a time-dependent worker-group effect (*p* = 0.340).

Grooming behaviours were analysed using generalized linear models (GLMs) fitted with the *glmmTMB* package, with colony included as a fixed effect to assess intercolonial differences among *L. flavus* colonies. Allogrooming was quantified as the cumulative duration of allogrooming events (in seconds) during 5-minute trials and analysed using a Tweedie model with a log link, which is appropriate for continuous, right-skewed data with many zero values (trials with no allogrooming observed). Self-grooming was quantified as a score per group and analysed using a beta-binomial model with a logit link, as the score represented the number of workers engaged in self-grooming out of 25 possible observations and showed overdispersion relative to a binomial distribution. Acidopore grooming was quantified as the number of acidopore grooming events per trial and analysed using a Poisson model, as residual diagnostics indicated no significant overdispersion and no evidence of zero inflation. For each grooming behaviour, differences among colonies were assessed by comparing models with and without the colony effect using LRTs. Model adequacy was evaluated using *DHARMa* residual diagnostics.

Intercolonial variation in the dynamics of necrophoresis was described from the number of corpses remaining inside the nest throughout the 4-hour observation period. Because the exact timing of corpse movements was recorded, complete corpse-removal trajectories could be reconstructed for each colony. However, as some corpses were occasionally retrieved by workers back into the nest, corpse removal was not an irreversible event, precluding the use of standard survival analyses. Corpse-removal dynamics were therefore summarized for each colony by calculating the area under the curve (AUC) of the number of corpses remaining in the nest over the first 2 hours, representing early corpse removal, and over the complete 4-hour observation period. For AUC calculation, the number of corpses remaining in the nest was considered constant between successive recorded movements. The maximum possible AUC value was 2400 for the 2-hour period (20 corpses × 120 minutes) and 4800 for the 4-hour period (20 corpses × 240 min), corresponding to no corpse removal during the respective observation period. Lower AUC values indicate faster corpse removal and shorter cumulative exposure of colony members to corpses.

Finally, the association between intercolonial differences in pathogen susceptibility and variation in prophylactic behaviours such as grooming and necrophoresis was evaluated. All correlations were assessed using Spearman correlation tests, with statistical significance evaluated using permutation-based inference (10 000 permutations) implemented in the package *coin*. For grooming analyses, colony-level pathogen susceptibility was estimated as the proportion of intranidal workers that died across the three infection assays (30 intranidal workers per colony in total), as grooming was quantified in intranidal workers. The associations between pathogen susceptibility and the median values of allogrooming, self-grooming and acidopore grooming were assessed, and *p*-values from these three correlations were adjusted for multiple testing using the Holm correction. For necrophoresis, colony-level pathogen susceptibility was estimated as the proportion of all workers that died across the three infection assays (60 workers per colony in total), and was correlated with corpse-removal AUC over the first 2 hours and over the complete 4-hour observation period. The *p*-values from these two correlations were adjusted for multiple testing using the Holm correction.

In order to confirm the independence of experimental colonies, all colony-level measures of pathogen susceptibility and sanitary behaviours were tested for spatial autocorrelation. Geographic coordinates of collection sites were projected into a metric coordinate system (UTM, WGS84 zone 31N) using the *sf* package. Spatial relationships among colonies were defined using a k-nearest-neighbour approach (*k* = 2) implemented in the *spdep* package, reflecting the linear arrangement of sampling sites along transects. Spatial autocorrelation was first evaluated using a global Moran's *I* test. When Moran's *I* was significant, local indicators of spatial association (LISA) were computed to identify potential clusters of neighbouring colonies with similar values. LISA *p*-values were adjusted for multiple testing using the Holm correction method. No significant global spatial autocorrelation was detected for any measure except allogrooming (Moran's *I* = 0.22, *p* = 0.040), for which LISA revealed no significant local clusters after Holm correction (supplementary material, table S2).

## 3. Results

### 3.1. Colony-specificity of pathogen susceptibility

Worker susceptibility to the entomopathogenic fungus *B. bassiana* significantly differed among *L. flavus* colonies (Cox model: *χ*² = 54.12, d.f. = 16, *p* < 0.001). Across the three infection assays, cumulative mortality varied widely among colonies, ranging from 5% in the most resistant colony to 42% in the most susceptible one. Mortality levels were consistent within each colony across repeated assays. Colony-level susceptibility was significantly repeatable over the three infection assays (ICC = 0.41 [95% CI: 0.11–0.70], *p* = 0.003) (figure 1), indicating that susceptibility to *B. bassiana* represents a stable trait at the colony level. In addition to intercolonial differences, pathogen susceptibility also varied within colonies between functional groups of *L. flavus* workers. Foragers were significantly more susceptible to *B. bassiana* than intranidal workers (Cox model: HR = 1.44 [95% CI: 1.12–1.86], *p* = 0.005), with mortality reaching 27.6% in foragers compared with 20.0% in intranidal workers by the end of the 12-day monitoring period (supplementary material, figure S1). Control groups exhibited very low mortality, with an overall cumulative mortality of 2.2% at the end of the experiments.

**Figure 1.**
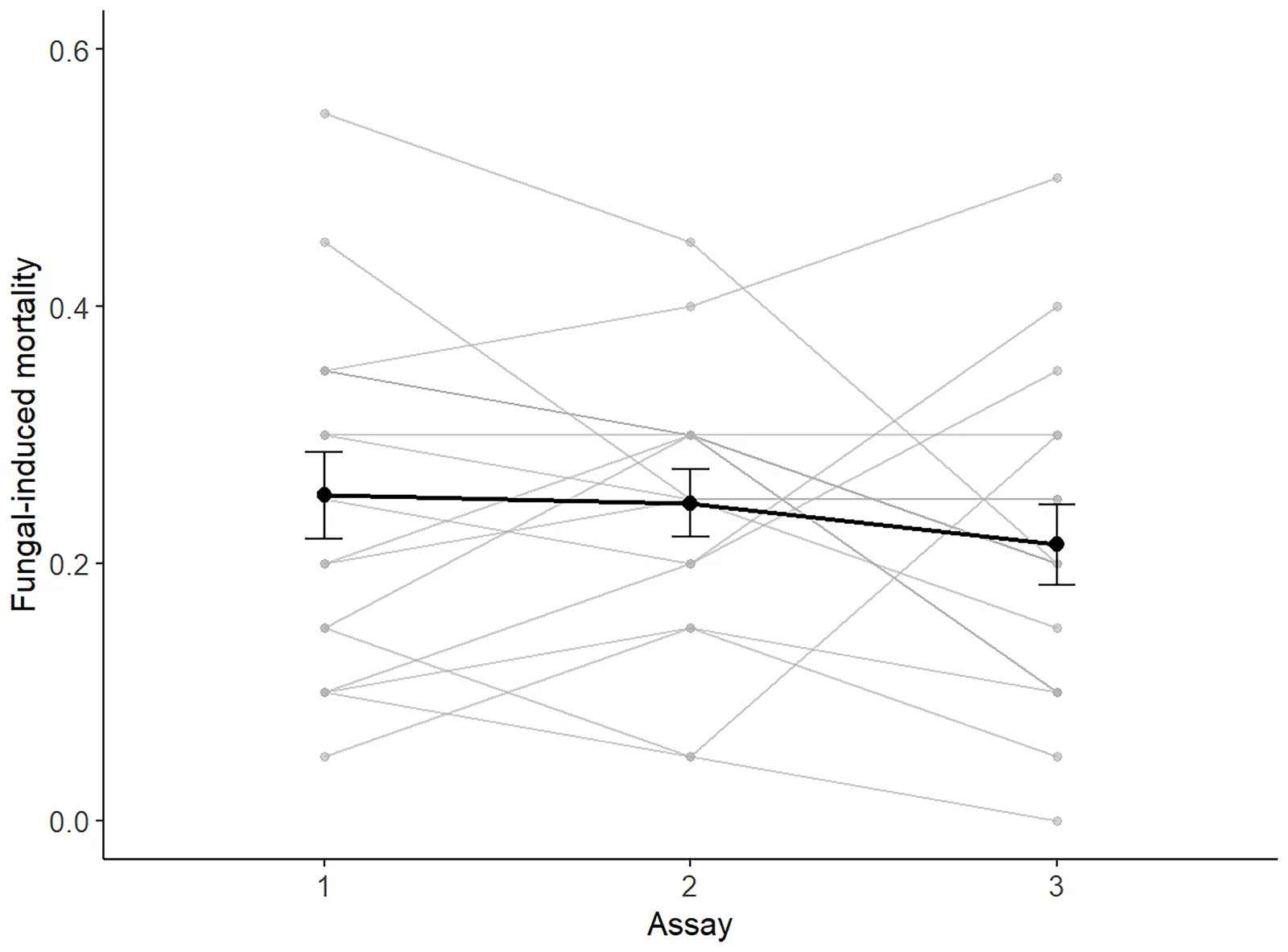
Colony-level repeatability of fungal-induced mortality across three infection assays in 17 colonies of *Lasius flavus*. Grey lines with points show mortality across the three assays for each colony. Black points indicate the mean mortality across colonies for each assay, with error bars representing the standard error. ICC = 0.41 [95% CI: 0.11–0.70], *p* = 0.003.

### 3.2. Intercolonial variation in prophylactic grooming behaviour

All sanitary grooming behaviours showed significant intercolonial variation among *L. flavus* colonies. Allogrooming varied markedly among colonies, with colony identity having a significant effect on the cumulative duration of allogrooming events (GLM: *χ*² = 38.95, d.f. = 16, *p* = 0.001). The median duration of allogrooming ranged from 0 seconds in three colonies (no allogrooming observed) to 265 seconds in the most active colony (supplementary material, figure S2a). Self-grooming also differed significantly among colonies (GLM: *χ*² = 29.33, d.f. = 16, *p* = 0.022), with median self-grooming scores ranging from 3 to 9 per colony (supplementary material, figure S2b). Likewise, acidopore grooming exhibited pronounced intercolonial variation, with colony identity strongly affecting the number of acidopore grooming events (GLM: *χ*² = 76.33, d.f. = 16, *p* < 0.001). Fifteen of the 17 tested colonies displayed few or no acidopore grooming events, whereas the remaining two colonies showed median values of four acidopore grooming events per trial (supplementary material, figure S2c). Together, these results revealed substantial intercolonial variation in prophylactic grooming behaviours.

### 3.3. Intercolonial variation in corpse removal

Corpse removal dynamics varied markedly among *L. flavus* colonies, with pronounced differences in both the timing and extent of corpse removal over the 4-hour observation period (figure 2). Following the introduction of 20 corpses into the nest, all colonies started to remove corpses, except for one colony that did not remove any corpses. Colonies differed in how rapidly necrophoresis was initiated. Latency to the first corpse removal event ranged from 11 to 240 minutes (the latter corresponding to one colony in which no corpse removal occurred), with a median latency of 30 minutes [21; 37]. After 1 hour, the number of corpses removed ranged from 0 to 15, with a median of 2 [1; 6] corpses out of 20. After 2 hours, the number of removed corpses increased but remained highly variable among colonies, with a median of 8 [2; 10] corpses. This marked variability persisted after 3 hours, when colonies had removed a median of 13 [3; 15] corpses. By the end of the experiment (4 hours), six colonies had removed nearly all corpses (≥18), whereas four colonies had removed few or none (≤5), with a median of 16 [6; 18] corpses removed. Consistent with this variation in removal dynamics, 2-hour AUC values ranged from 1018 to 2400, while 4-hour AUC values ranged from 1258 to 4800 across colonies. Overall, these observations highlight substantial intercolonial variation in necrophoretic behaviour.

**Figure 2.**
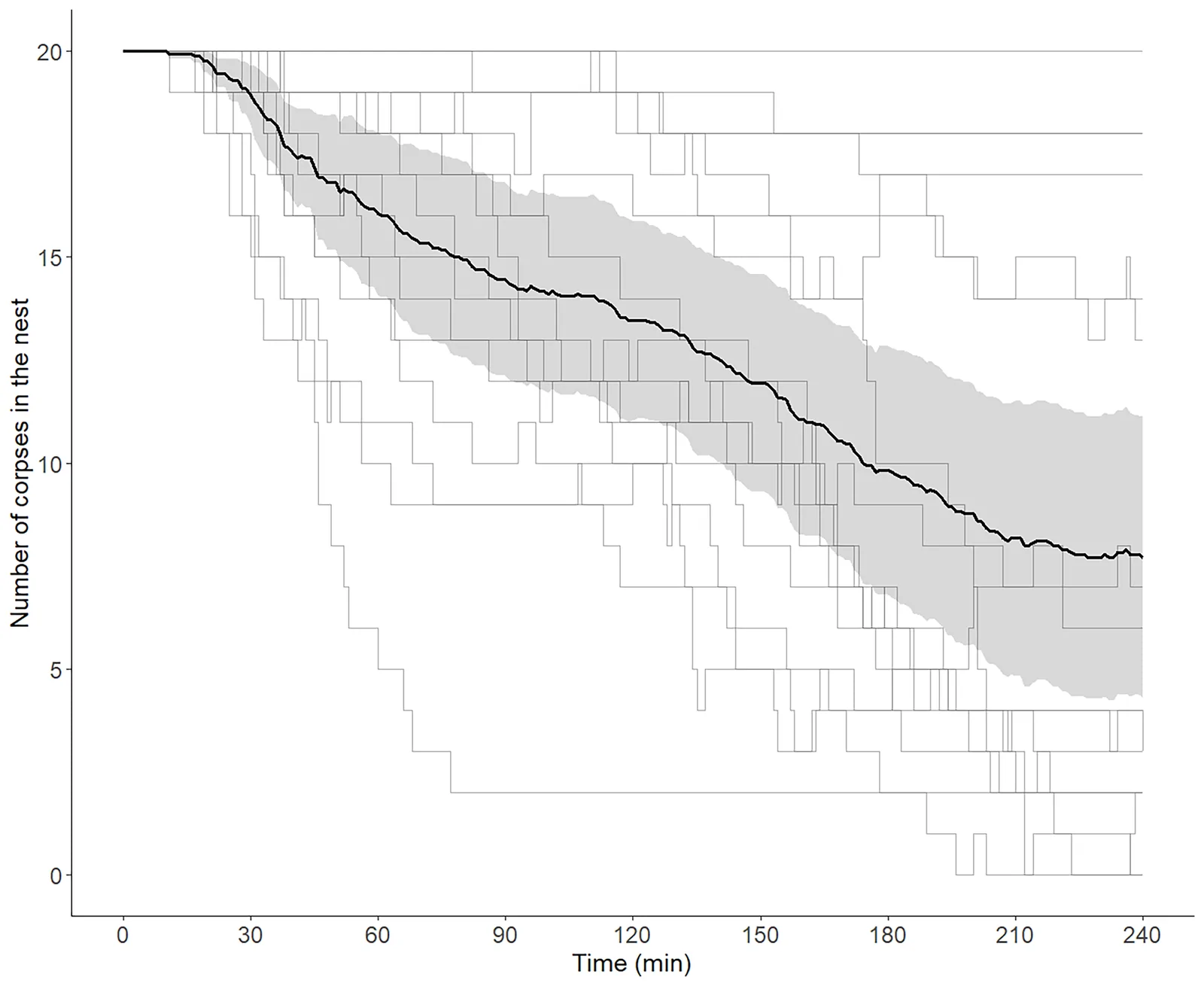
Intercolonial variation in the dynamics of necrophoresis in *Lasius flavus*. Thin grey lines show the dynamics of corpse removal for each colony (*n* = 17), expressed as the number of corpses remaining inside the nest over the 4-hour experiment. The thick black line represents the mean number of corpses remaining in the nest across colonies at each time point, and the shaded band indicates the 95% confidence interval around the mean.

### 3.4. Relationships between pathogen susceptibility and prophylactic behaviours

We found that intercolonial variation in pathogen susceptibility was not associated with variation in grooming behaviours. Intranidal worker mortality was not significantly correlated with median values of allogrooming (Spearman test: *ρ* = –0.26, *p* = 0.608), self-grooming (Spearman test: *ρ* = –0.35, *p* = 0.504) or acidopore grooming (Spearman test: *ρ* = –0.15, *p* = 0.608). For necrophoresis, overall worker mortality showed a negative association with 2-hour corpse-removal AUC. More susceptible colonies tended to remove corpses more rapidly during the early phase of necrophoresis, but this association was not statistically significant after Holm correction (Spearman test: *ρ* = –0.51, unadjusted *p* = 0.035, Holm-adjusted *p* = 0.070) (figure 3). Overall worker mortality was not significantly associated with 4-hour corpse-removal AUC (Spearman test: *ρ* = –0.38, *p* = 0.130) (supplementary material, figure S3).

**Figure 3.**
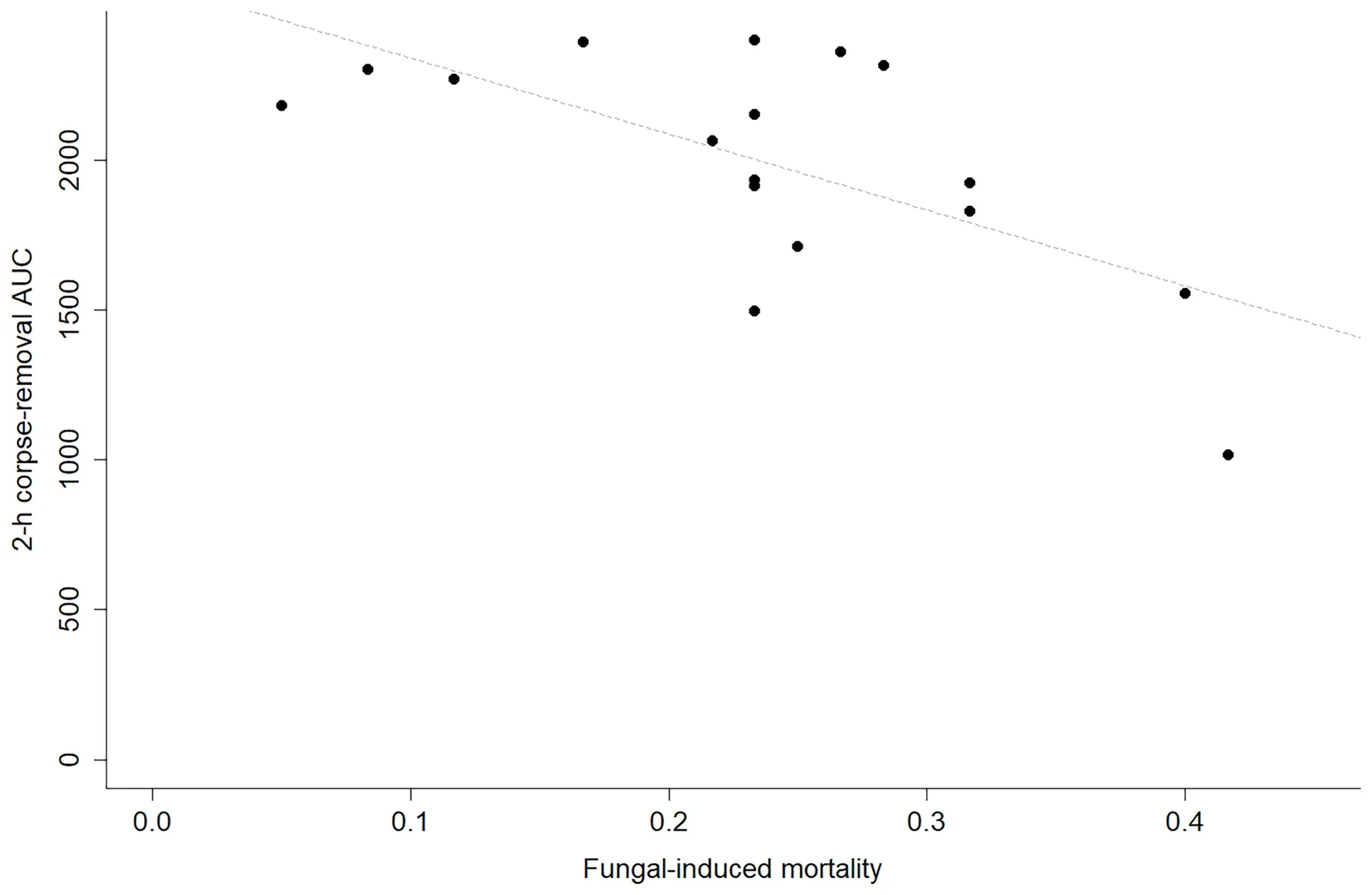
Relationship between pathogen susceptibility and early corpse removal among *Lasius flavus* colonies. Each point represents one colony (*n* = 17). The *x*-axis shows the proportion of workers killed by *Beauveria bassiana* across three infection assays (60 workers per colony), and the *y*-axis shows corpse-removal AUC, calculated from the number of corpses remaining inside the nest over the first 2 hours of the observation period. Lower AUC values indicate faster corpse removal. Spearman test: *ρ* = –0.51, unadjusted *p* = 0.035, Holm-adjusted *p* = 0.070.

## 4. Discussion

We found that *L. flavus* colonies consistently differed in their susceptibility to the entomopathogenic fungus *B. bassiana*. Across repeated infection assays, some colonies repeatedly experienced high worker mortality, whereas others remained markedly more resistant to fungal infection. This finding confirms our previous study reporting intercolonial variation in survival following fungal exposure in this ant species [31]. It further demonstrates that these differences remain consistent across several infection assays performed on different groups of workers from the same colonies. Susceptibility to *B. bassiana* therefore appears to represent a consistent colony-level trait. Comparable intercolonial differences in susceptibility to fungal pathogens have been reported in other social insects, including ants, termites and honeybees [28,36,37]. One possible explanation for the consistency in pathogen susceptibility within colonies is the influence of genetic background on individual resistance [1]. In our study, workers were exposed individually to the pathogen under controlled conditions, excluding the contribution of social defences provided by nestmates. Differences in susceptibility may therefore reflect variation in multiple components of individual defence. These include genetically influenced physiological traits affecting host resistance, such as cuticular properties or immune responsiveness, but also behavioural responses induced following pathogen exposure, such as enhanced grooming. Indeed, grooming can reduce fungal contamination by removing conidia from the body surface before infection [7,38], and our previous study showed significant intercolonial variation in pathogen-induced self-grooming and acidopore grooming in *L. flavus* following exposure to *B. bassiana* [31]. Such variation could reflect differences among colonies in workers’ sensitivity or response thresholds to pathogen-related cues, thereby affecting their ability to limit infection alongside physiological defences. A genetic component of individual grooming responses has been identified in honeybees [39,40], suggesting that variation in the induction of behavioural defences could also be heritable. Since nestmates are more genetically similar to one another than to workers from different colonies, genetically influenced variation in these individual defences could contribute to the consistent differences in pathogen susceptibility observed among colonies. This pattern may be particularly pronounced in monogynous and monandrous colonies, a social structure relatively common in *L. flavus* [41], where relatedness among workers is expected to be especially high. Conversely, greater genetic diversity among workers could improve colony resilience to pathogens by increasing the variability of resistance phenotypes within colonies [42].

In addition to differences in pathogen susceptibility, we observed significant intercolonial variation in the expression of self-grooming, allogrooming and acidopore grooming behaviours. In our study, these grooming behaviours were quantified in the absence of pathogen exposure in order to assess their prophylactic expression. However, none of these grooming behaviours was correlated with worker susceptibility to fungal infection, suggesting that these components of disease defence may vary independently among colonies. This may be explained, at least partly, by the fact that self- and allogrooming in ants are involved in multiple functions, including the removal of infectious particles from the body surface, the cleaning of sensory organs and the redistribution of cuticular compounds contributing to colony odour homogenization [8]. Consequently, variation in the expression of self- and allogrooming likely reflects multiple functions and benefits beyond pathogen prophylaxis. By contrast, acidopore grooming is more specifically associated with antimicrobial defence in Formicinae ants, as workers can use formic acid secretions both to disinfect their body surface and to sanitize the digestive tract [9,43]. Acidopore grooming nevertheless remained relatively rare under our experimental conditions. This low expression may reflect the absence of direct infection cues in our assays, suggesting that acidopore grooming is more strongly expressed in response to immediate pathogenic threats [9].

Necrophoresis constitutes another key sanitary behaviour in social insects, contributing to colony hygiene through the elimination of dead individuals and other potentially hazardous waste from the nest [17]. Although corpse removal is often viewed as a response to infectious corpses, the disposal of non-infectious corpses also represents a prophylactic behaviour by limiting the future accumulation of microbial hazards within the nest. In our experiments, all *L. flavus* colonies except one initiated corpse removal, but the dynamics of necrophoresis varied markedly among colonies. Several factors may influence the speed of corpse removal. First, removal can depend on corpse-related cues, which change following death. These include the accumulation of oleic and linoleic fatty acids on the cuticle of dead individuals [14,35] and pathogen-associated cues from infectious items [44,45]. Because the time since death can strongly influence necrophoretic responses [35,46,47], all corpses were standardized at 48 hours post-mortem before being introduced into the nests. Differences in corpse age can therefore be ruled out as an explanation for the variation in corpse-removal dynamics observed among colonies under our experimental conditions. Second, colony-level organizational factors may shape necrophoresis dynamics. Corpse removal is often performed by a subset of workers, suggesting some degree of task specialization in waste management [16,32,48,49]. Differences among colonies in the number of workers specialized in necrophoresis, or in their response thresholds to corpse-related cues, could therefore contribute to variation in the speed and extent of corpse removal. In our study, the corpses introduced into nests were not infectious, which may have reduced the urgency of removal. Under such low-risk conditions, differences among colonies in whether and how rapidly workers respond to corpse-related cues may become particularly apparent. By contrast, exposure to highly infectious material may induce a more generalized sanitary response across workers, as observed in *Myrmica rubra* exposed to sporulating corpses [32]. Such a broader sanitary response may reduce intercolonial variation in necrophoresis by promoting rapid corpse removal across colonies.

Beyond corpse pathogenicity, the social context of a colony can also influence sanitary and collective behaviours in ants. In particular, brood can enhance waste-removal responses [45], while queen presence and group size can modify colony organization and task performance [50–52]. Our experiments were conducted with standardized, queenless and broodless groups of equal size. Future studies could therefore examine whether necrophoretic responses and the magnitude of intercolonial variation differ under more complex social contexts.

We observed a tendency for a positive relationship between pathogen susceptibility and corpse removal efficiency, with colonies that removed corpses more rapidly during the early phase of necrophoresis also tending to exhibit higher worker mortality following exposure to *B. bassiana*. One possible interpretation is that this pattern reflects a compensatory relationship among defence components, such that colonies characterized by higher worker susceptibility also exhibit stronger expression of prophylactic sanitary behaviours. Because individual immune defences can be costly, selection may favour greater reliance on other components of disease defence, such as the prophylactic removal of potential sources of infection. However, for such a compensatory relationship to evolve, the traits involved would need to have a heritable component. In ants, genetic effects have been demonstrated for both pathogen resistance [42] and workers’ propensity to perform waste-management tasks [53,54]. Nevertheless, whether necrophoretic behaviour has a heritable basis remains unknown. Furthermore, previous studies examining relationships between individual defence and sanitary behaviours in social insects have produced inconsistent results. Cassidy *et al.* [28] reported that ant colonies exhibiting higher pathogen-induced mortality performed more efficient prophylactic corpse removal. By contrast, Scharf *et al.* [55] found no association between corpse removal and immune defence in ants, and Harpur *et al.* [30] found no relationship between brood removal behaviour and innate immunity in honeybees. These contrasting findings suggest that associations between individual defence and prophylactic sanitary behaviours are not consistently observed across social insects or across different components of disease defence.

The potential sanitary benefit of faster corpse removal, particularly as a mechanism that could compensate for higher pathogen susceptibility, should also be considered with caution. The positive association between pathogen susceptibility and necrophoresis efficiency was mainly apparent during the early phase of necrophoresis. Because the corpses introduced into nests were not infectious, faster removal during this initial period may not provide a major sanitary benefit for the colony. Furthermore, previous work has shown that ant colonies can tolerate non-infectious corpses in the nest for several days, and that even severe impairment of corpse removal has only limited effects on worker and brood survival [56].

An alternative explanation for this relationship may lie in demographic and organizational differences among colonies, which could simultaneously influence worker susceptibility and necrophoresis without requiring a genetic association between these traits. In social insects, foraging is typically performed by older workers as part of age-related division of labour, whereas brood care and other intranidal tasks are generally performed by younger individuals [57,58]. At the same time, the foraging role may itself be associated with weaker immune defences [59], while immune performance may also decline with age, a phenomenon commonly referred to as immunosenescence and reported in several social insects [60–62]. Consistent with these patterns, foragers in our study were more susceptible to fungal infection than intranidal workers, confirming the results of our previous work on *L. flavus* [31]. Corpse removal and waste management are generally performed by workers engaged in external tasks, often corresponding to foraging individuals [16,32]. Colonies differing in worker age structure or task allocation could therefore exhibit both higher pathogen susceptibility and more efficient necrophoresis. The association between higher pathogen susceptibility and more efficient corpse removal may therefore arise as a by-product of demographic or organizational differences among colonies rather than from evolutionary compensation between these components of disease defence.

Overall, our results reveal pronounced colony-level variation in both pathogen susceptibility and prophylactic sanitary behaviours in *L. flavus*. Colonies differed markedly in their susceptibility to fungal infection as well as in the expression of grooming and necrophoresis, highlighting substantial heterogeneity in multiple components of disease defence within a single species. However, our results provide little support for a compensatory relationship between pathogen susceptibility and prophylactic behaviour. Grooming behaviours were not related to worker susceptibility, while colonies experiencing higher pathogen-induced mortality tended to remove corpses more rapidly only during the early phase of necrophoresis. Rather than reflecting evolutionary compensatory processes, this relationship may instead arise from demographic and organizational differences among colonies, including variation in worker age structure and task allocation. More broadly, these findings emphasize the importance of considering colony organization and demographic structure when interpreting relationships among different components of disease defence in social insects.

## Supplementary Material



## Data Availability

The dataset and R script supporting this study are provided as supplementary material [63].
