# Supplementary material for: Prophylaxis in ant societies: a social response to pathogen vulnerability?
Source: R Soc Open Sci. 2026 Nov 11;13(11):261179. doi: 10.1098/rsos.261179 (PMC13750941; doi:10.1098/rsos.261179)
Supplement: Supplementary file 1 [file rsos.261179.zip › rsos.261179/rsos261179_si_001.pdf]

Prophylaxis in ant societies: a social response to pathogen vulnerability?

Authors: Romain WILLEPUT & Claire DETRAIN

Unit of Social Ecology, Université Libre de Bruxelles, Brussels, Belgium

Corresponding author: romain.willeput@ulb.be

RW: 0009-0006-1636-736X

CD: 0000-0002-3068-1877

Royal Society Open Science

DOI: 10.1098/rsos.261179

Supplementary material

Table S1. Geographic coordinates of the 17 *Lasius flavus* colonies used in the study. Coordinates are provided in decimal degrees (WGS84).

Colony	Latitude (°N)	Longitude (°E)
C1	51.19808	2.93990
C2	51.20100	2.93706
C3	51.20086	2.93703
C4	51.20065	2.93663
C5	51.20041	2.93621
C6	51.20160	2.93676
C7	51.20040	2.93608
C8	51.20030	2.93586
C9	51.20020	2.93566
C10	51.20016	2.93553
C11	51.20008	2.93535
C12	51.20001	2.93515
C13	51.19993	2.93496
C14	51.19976	2.93445
C15	51.19970	2.93428
C16	51.19961	2.93398
C17	51.19951	2.93386

Table S2. Spatial autocorrelation analyses of colony-level pathogen susceptibility and prophylactic sanitary behaviours using Moran's I. Local Indicators of Spatial Association (LISA) were computed only when the Moran's I test was significant.

**Proxy**	**Moran's I**	***p*-value (Moran)**	**LISA**
Susceptibility	-0.18	0.72	/
Allo-grooming	0.22	0.04	No local cluster
Self-grooming	-0.28	0.84	/
Acidopore-grooming	-0.22	0.79	/
Necrophoresis (AUC 2 h)	-0.03	0.43	/
Necrophoresis (AUC 4 h)	-0.17	0.70	/

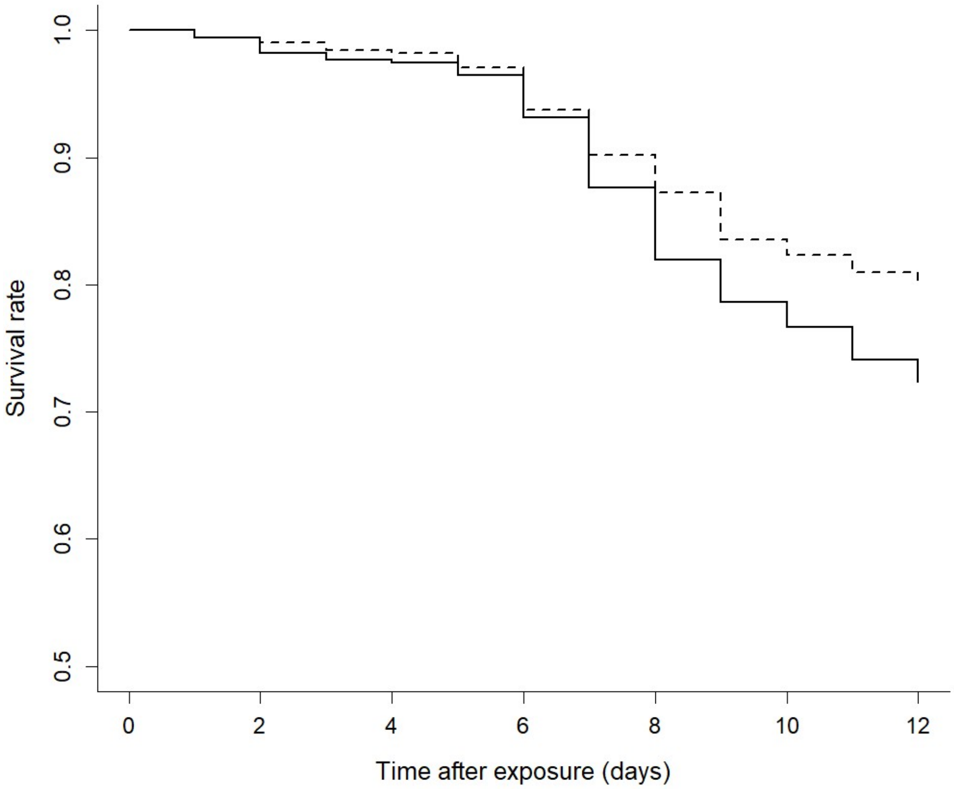

Figure S1. Kaplan-Meier survival curves of foragers and intranidal workers of *Lasius flavus* after exposure to *Beauveria bassiana*. Solid and dashed lines represent foragers and intranidal workers, respectively. Cox model: HR = 1.44 [95% CI: 1.12–1.86], p = 0.005, n = 510 workers per functional group.

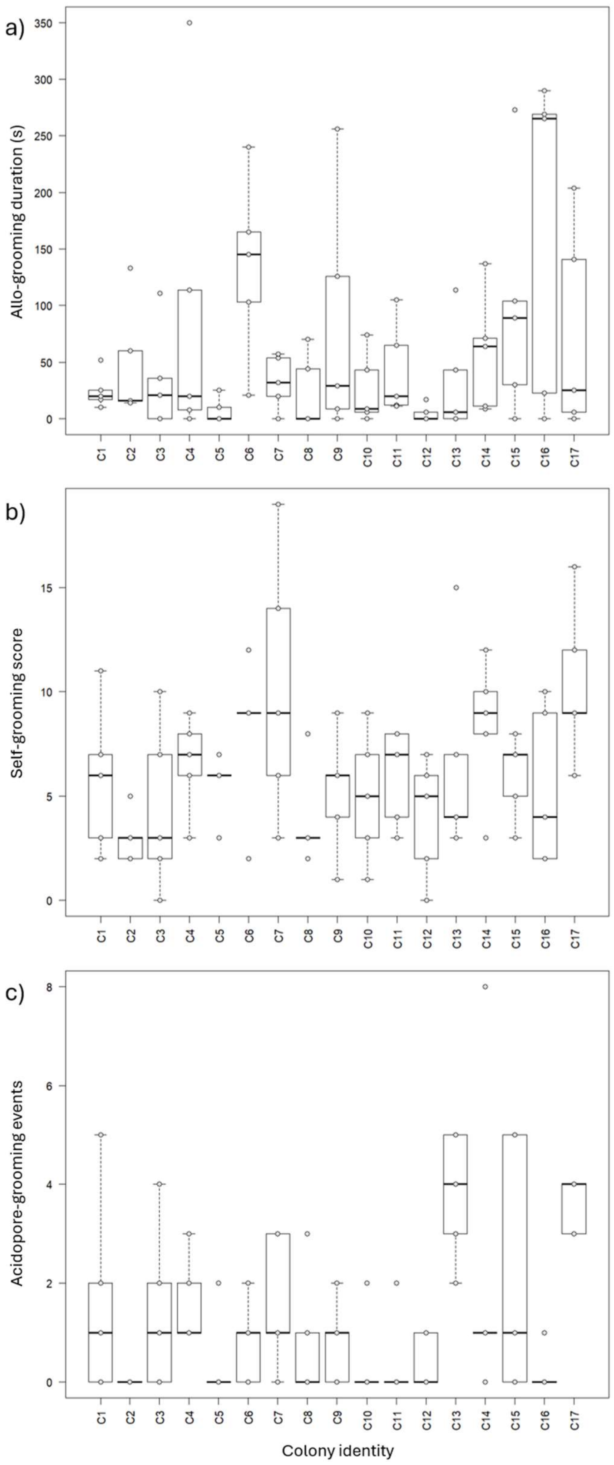

Figure S2. Intercolonial variation in prophylactic grooming behaviours in *Lasius flavus* workers (n = 17 colonies). For each colony, grooming behaviours were quantified in five groups of five intranidal workers observed for 5 min. **(a)** Allo-grooming expressed as the cumulative duration (s) of allo-grooming events per group (GLM: χ^2 = 38.95, df = 16, p = 0.001). **(b)** Self-grooming score per group, calculated as the summed number of workers engaged in self-grooming during the last 10 s of each minute (GLM: χ^2 = 29.33, df = 16, p = 0.022). **(c)** Number of acidopore-grooming events per group (GLM: χ^2 = 76.33, df = 16, p < 0.001).

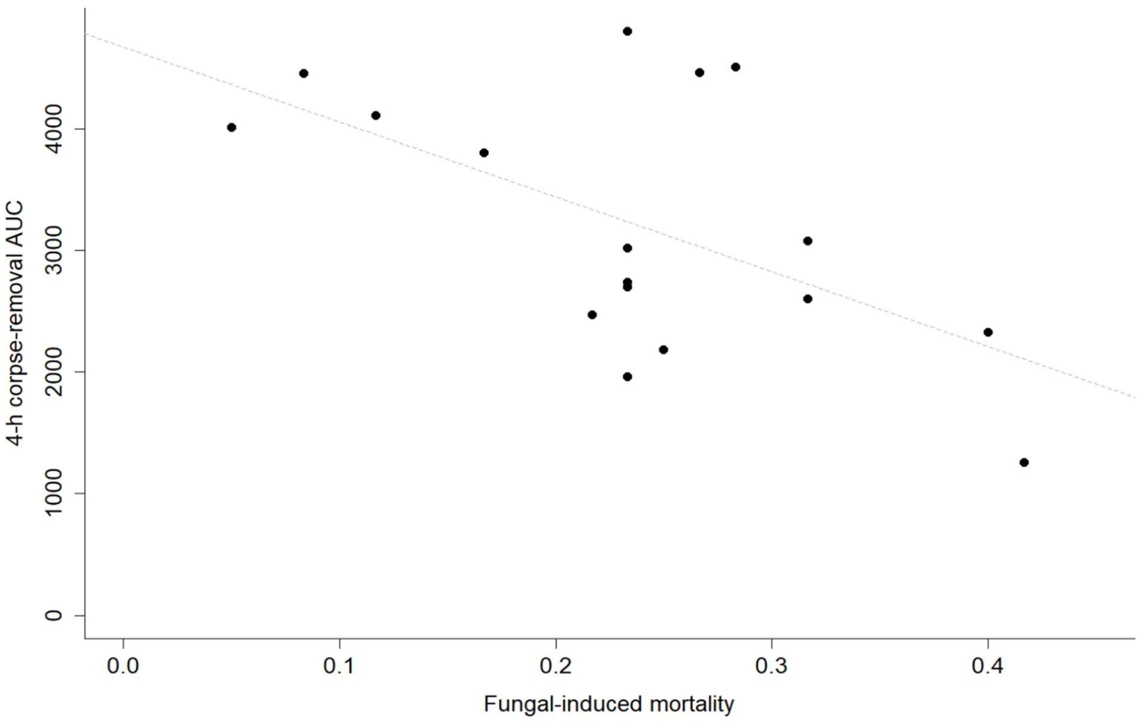

Figure S3. Relationship between pathogen susceptibility and corpse removal among *Lasius flavus* colonies. Each point represents one colony (n = 17). The x-axis shows the proportion of workers killed by *Beauveria bassiana* across three infection assays (60 workers per colony), and the y-axis shows corpse-removal AUC, calculated from the number of corpses remaining inside the nest over the 4-h observation period. Lower AUC values indicate faster corpse removal. Spearman test: $\rho = -0.38$, $p = 0.130$.
